# Plasticity in growth of farmed and wild Atlantic salmon: is the increased growth rate of farmed salmon caused by evolutionary adaptations to the commercial diet?

**DOI:** 10.1186/s12862-016-0841-7

**Published:** 2016-12-01

**Authors:** Alison Catherine Harvey, Monica Favnebøe Solberg, Eva Troianou, Gary Robert Carvalho, Martin Ian Taylor, Simon Creer, Lise Dyrhovden, Ivar Helge Matre, Kevin Alan Glover

**Affiliations:** 1 Molecular Ecology and Fisheries Genetics Laboratory, School of Biological Sciences, Deiniol Road, Bangor University, Bangor, LL57 2UW UK; 2 Institute of Marine Research, P.O. Box 1870, Nordnes, NO-5817 Bergen Norway; 3 School of Biological Sciences, University of East Anglia, NR4 7TJ Norwich, UK; 4 Sea Lice Research Centre, Department of Biology, University of Bergen, Bergen, Norway

**Keywords:** Domestication, Farm escapes, Genetic interaction, Hybridisation, Reaction norms, Survival, Salmonids, Pellets, Natural diet, Feed utilisation, Appetite

## Abstract

**Background:**

Domestication of Atlantic salmon for commercial aquaculture has resulted in farmed salmon displaying substantially higher growth rates than wild salmon under farming conditions. In contrast, growth differences between farmed and wild salmon are much smaller when compared in the wild. The mechanisms underlying this contrast between environments remain largely unknown. It is possible that farmed salmon have adapted to the high-energy pellets developed specifically for aquaculture, contributing to inflated growth differences when fed on this diet. We studied growth and survival of 15 families of farmed, wild and F1 hybrid salmon fed three contrasting diets under hatchery conditions; a commercial salmon pellet diet, a commercial carp pellet diet, and a mixed natural diet consisting of preserved invertebrates commonly found in Norwegian rivers.

**Results:**

For all groups, despite equal numbers of calories presented by all diets, overall growth reductions as high 68 and 83%, relative to the salmon diet was observed in the carp and natural diet treatments, respectively. Farmed salmon outgrew hybrid (intermediate) and wild salmon in all treatments. The relative growth difference between wild and farmed fish was highest in the carp diet (1: 2.1), intermediate in the salmon diet (1:1.9) and lowest in the natural diet (1:1.6). However, this trend was non-significant, and all groups displayed similar growth reaction norms and plasticity towards differing diets across the treatments.

**Conclusions:**

No indication of genetic-based adaptation to the form or nutritional content of commercial salmon diets was detected in the farmed salmon. Therefore, we conclude that diet alone, at least in the absence of other environmental stressors, is not the primary cause for the large contrast in growth differences between farmed and wild salmon in the hatchery and wild. Additionally, we conclude that genetically-increased appetite is likely to be the primary reason why farmed salmon display higher growth rates than wild salmon when fed *ad lib* rations under hatchery conditions. Our results contribute towards an understanding of the potential genetic changes that have occurred in farmed salmon in response to domestication, and the potential mechanisms underpinning genetic and ecological interactions between farmed escapees and wild salmonids.

**Electronic supplementary material:**

The online version of this article (doi:10.1186/s12862-016-0841-7) contains supplementary material, which is available to authorized users.

## Background

Aquaculture is now the fastest growing food sector in the world, supplying over half of the world’s fish protein [1]. One of the most economically important aquaculture species is the Atlantic salmon (*Salmo salar* L.), an anadromous salmonid fish which is endemic to rivers on the west and east coasts of the Atlantic Ocean in the Northern hemisphere [2]. Atlantic salmon farming originated in Norway in the very late 1960s, and in recent years the industry has grown worldwide to include commercial efforts in a number of countries both within and beyond the species natural range, for example: Chile, Scotland (UK) and Canada [1]. Current global production of Atlantic salmon exceeds two million tonnes, over half of which is produced in Norway alone [3].

Selective breeding programs began shortly after the first commercial farming efforts commenced in Norway, and current strains of salmon have undergone up to twelve or more generations of directional selection for traits of commercial importance [4, 5]. The initial breeding goals for salmon aquaculture were to increase growth rate and subsequently to delay sexual maturation, and that soon expanded to include disease resistance, flesh colour and body composition [6]. The genetic gain for growth-rate in salmon has been estimated at 10–15% per generation [4], and selection has thus increased growth rates of farmed salmon by several-fold compared to wild conspecifics under hatchery conditions [7–9]. It has also been demonstrated that selection for increased growth has indirectly increased appetite and feed conversion efficiency (FCE) [10–13], although on a more modest scale.

In intensive aquaculture, feed is continuously provided in the form of high-energy pellets, and is formulated to provide the fish with all their species-specific nutritional requirements while maximising feed utilisation. In commercial salmon aquaculture, one of the highest operating costs is feed, which can be as much as 60% of the cost of production [13]. As the understanding of the nutritional requirements of Atlantic salmon has increased, commercial diets have been continuously refined to more closely meet energy and nutrient needs while striving to utilise more cost-effective ingredients [14]. Salmon are carnivorous, requiring diets that are high in protein and contain essential fatty acids [15]. Traditionally these nutrients were obtained by including large amounts of fish meal and fish oil in salmonid diets. However, in light of sustainable intensification, the inclusion of marine sources of proteins and lipids in salmon diets is slowly declining in favour of plant substitutes [16]. Thus, the commercial salmon diet does not only deviate from the wild diet in terms of form (i.e., pellet vs. natural prey), but also in terms of energy content and nutritional profile. The natural diets of wild fish can vary considerably in terms of type and form of prey, density of calories and nutrient composition. In freshwater habitats juvenile salmon typically feed on drift and benthic invertebrates, the availability of both will depend on the specific habitat characteristics such a flow rate and substrate [17].

Domestication involves adaptation to a captive environment, which is very different to the natural environment experienced by wild conspecifics. These differences can lead to both phenotypic and behavioural differences between domesticated and wild individuals [18, 19], and domestication-mediated genetic changes may occur within a single generation [20]. The changes are a result of direct and indirect responses to artificial selection and relaxed natural selection, and the low mortality associated with the domestic environment may result in phenotypes persisting where they would not have persisted in the wild [18, 19, 21]. In addition to a moderately increased FCE linked to significantly higher growth rates, farmed salmon also exhibit changes relative to wild salmon for other feeding related traits such as increased appetite [10], growth hormone (GH) [22] and insulin-like growth factor (IGF-I) [23]. It is possible that generations of selection for fast growing fish have resulted in farmed salmon that are adapted to the form and high calorie content of salmon pellets. Farmed salmon have been fed using a pelleted diet since commercial salmon aquaculture began, while in the wild, fish are opportunistic feeders and actively seek out feed, typically varying their diet in order to obtain the essential nutrients required for growth [17]. Therefore, adaptation to commercial salmon pellets may partly explain why there are such large growth differences observed between farmed and wild salmon under farming conditions [7–9] with considerably less differences observed under natural conditions [24–26].

Exploring whether indirect selection for feeding related traits has influenced growth and survival in domestic and wild conspecifics will advance our knowledge of the changes elicited by domestication of Atlantic salmon. In turn, this will also help shed light on the potential evolutionary consequences of farmed escapees where they have been demonstrated to interbreed wild salmon populations [27–29]. Therefore, we investigated the growth and survival of farmed, wild and F1 hybrid Atlantic salmon offspring fed three contrasting diets within the hatchery using a common garden experimental design. The overall aim was to investigate whether over ten generations of selective breeding in farmed salmon has resulted in the indirect selection for adaptation to commercial salmon diets, thus explaining why farmed salmon are able to outgrow wild salmon by large ratios in the hatchery, but not in the wild [7, 9, 25]. Specifically, we hypothesised that if farmed salmon are genetically adapted to the nutritional content or form of the pelleted salmon diets then they would not be able to maintain their large relative growth difference over wild salmon when fed a commercial pelleted diet of unfamiliar nutritional content, nor when fed a diet whose form resembles a natural diet.

## Methods

### Experimental crosses

The farmed, wild and F1 hybrid families were produced in November 2013 (week 46) at the Matre Research station, Institute of Marine Research (IMR), Norway. Atlantic salmon originating from the commercial Mowi strain and wild Atlantic salmon caught in the river Etne (59°40’N, 5°56’E), were used to produce five pure farmed, five pure wild, and five F1 hybrid families (Additional file 1).

The Mowi strain is the oldest Norwegian domestic salmon strain [30]. The Mowi strain was originally established from salmon populations in rivers along the west coast of Norway in the 1960s, with main contributions from the River Bolstad and River Årøy [31]. The strain has been primarily selected for, among other traits, increased growth rate and has undergone over ten generations of selective breeding. As a consequence, offspring of Mowi farmed salmon display significantly higher growth rates under standard hatchery conditions in comparisons with the offspring of wild salmon [7–9]. However, in the wild, this farmed strain only displays slightly higher growth rates than wild conspecifics [25].

The salmon stock in the River Etne, located in south-west Norway, is the largest population within its fjord system Hardangerfjorden; the fourth longest fjord in the world and second longest in Norway. Wild adult broodstock were collected by angling in the River Etne in the autumn of 2013, transferred to the local hatchery and held until the stripping of gametes. A recent study of temporal genetic stability of salmon population across many Norwegian rivers revealed that Etne had not undergone any significant genetic change with time [28]. Growth patterns on fish scales were read on individuals in order to ensure that they were indeed born in the wild and were not farmed escapees [32].

The F1 hybrid fish were produced by crossing farmed females and wild males (Mowi ♀ x Etne ♂). The five hybrid families were thus maternal and paternal half-siblings with the farmed and wild families, respectively. All 15 families were incubated at ambient water temperature in single-family units until the eyed-egg stage.

### Experimental design & rearing conditions

Eyed eggs from families were sorted into hatchery trays representing the replicate treatments in week 5 of 2014. Each replicate treatment consisted of 30 eggs per family of each group, yielding 450 eggs in each of six replicates (two per treatment). In week 18 the hatched and ready-to-start feeding fry were transferred to six identical tanks (1.5 m^3^, ambient water temperature ranging from 4.5 to 14.6 °C with an average of 8.6 °C). The diet treatments were initiated when feeding commenced in week 18 of 2014. In 2014, the average water temperature in the Etne River was 14.6 °C with a range of 7.4 to 23.04 °C during the experimental period of the present study.

**Fig. 1:**
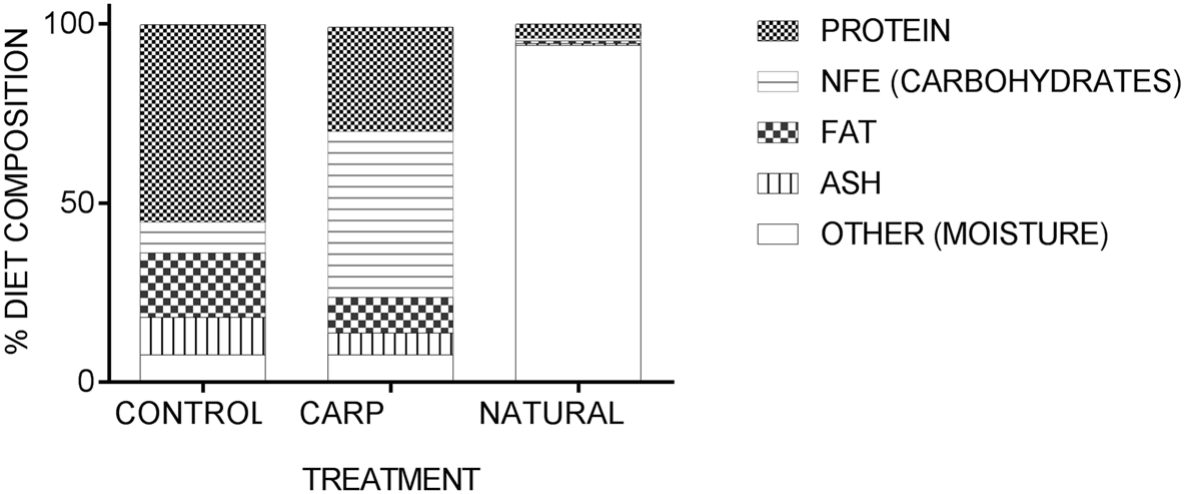
Stacked graph showing the average proportions of the main nutritional contents of each diet. Diet treatments: control, carp and natural. A more detailed description of the diet components is presented in Additional file 1

The control treatment consisted of a diet of commercial pelleted salmon feed, Skretting Nutra, which has a high protein and lipid content, with a low carbohydrate content. The carp treatment consisted of a commercial pelleted carp diet, Skretting Coarse Fish, which has a high level of carbohydrates and a lower protein and lipid content than the control diet. The natural treatment was composed of a combination of different frozen organisms which are typically present in the rivers of Norway; namely, a mix of freshwater copepods Cyclopidae *Cyclops*, water fleas Daphniidae *Daphnia* and insect larvaes; black mosquito larvae Culicidae and glassworms, i.e., transparent larvae of the phantom midge Chaoboridae *Chaoborus*. The three treatments are from here on referred to as the control, carp and natural treatments. Pellet sizes for the control and carp diets were adjusted according to the manufacturer’s feed table for the commercial salmon feed as the fish grew throughout the experiment. To obtain similar sized pellets for the control and carp diet, carp pellets were crushed and sieved (500 μm, 700 μM and 1 mm filter). Insects in the natural treatment were weighed and thawed before they were fed to the fish. The percentage of each organism within the natural diet treatment varied manually throughout the experiment to compensate for the growth of the fish, with smaller insects given in higher amounts at the start. All treatments received the same total calorific value each day, and feed was provided in excess for all treatments. The fish were fed for 12 h, and it was ensured that the calorific value of the treatments matched the total caloric value of a full ration of commercial salmon feed (5% of the fish dry weight/day) in order to eliminate competition effects. Non-eaten food was removed from the natural treatment replicates each day, before a new daily feeding cycle was initiated. The fish were kept on a 24-h photoperiod from transfer to tanks until experiment termination. During the experimental period, there was a non-biological mortality incident in one of the natural treatment replicates. However, both relative survival and growth at the family level was observed to be stable between replicates in this treatment, indicating that this mortality event did not unduly influence the results of this study. Potential variation in growth and survival between replicated tanks were, nonetheless, statistically controlled for during analysis. For an overview of the experimental design see Table 1. See Fig. 1 for a simple representation of the average contents of each diet and Additional file 1 for detailed nutritional contents of each diet.

**Table 1:** Overview of experimental design

Treatment	Control		Carp		Natural	
	Tank 1	Tank 2	Tank 3	Tank 4	Tank 5	Tank 5
Initial number per tank	15 Families:	15 Families:	15 Families:	15 Families:	15 Families:	15 Families:
	5 farmed	5 farmed	5 farmed	5 farmed	5 farmed	5 farmed
	5 F1 hybrid	5 F1 hybrid	5 F1 hybrid	5 F1 hybrid	5 F1 hybrid	5 F1 hybrid
	5 wild	5 wild	5 wild	5 wild	5 wild	5 wild
	30 eggs per family	30 eggs per family	30 eggs per family	30 eggs per family	30 eggs per family	30 eggs per family
	*n* = 450	*n* = 450*	*n* = 450	*n* = 450	*n* = 450	*n* = 450
Sampled	*n* = 422	*n* = 423	*n* = 290	*n* = 328	*n* = 215	*n* = 306

The ratios of Atlantic salmon families within each genetic group, the total number of fish in each replicate and the final number of surviving fish sampled from each replicated diet treatment are indicated. *One control replicate contained 451 eggs, as at the time of sorting one family was accidentally allocated one extra egg. The total number of fish sampled at experiment termination was 1984 as the numbers indicate here

### Sampling, genotyping and family assignment

The experiment was terminated in week 36 of 2014, when fish in all tanks were euthanised following standard guidelines with an overdose of Finquel® Vet anaesthetic (ScanVacc, Årnes, Norway). The fish were measured for wet weight and fork length, and a fin clip was taken from each and stored in individually labelled tubes filled with 100% ethanol for DNA analysis. A total of 1984 individuals were sampled (Table 2).

**Table 2:** Average weights and mortality of farmed, wild and hybrid Atlantic salmon within each replicate and treatment

Treatment	Origin	Tank	Initial n	Final n	Mortality		W (g)				Pooled W (g)			Pooled Mortality
					n	%	Mean	Median	SD	n	Mean	Median	SD	%
Control	Farm	1	150	138	9	6%	30.08	31.00	6.85	276	29.75	30.50	6.52	8.00
		2	150	138	11	7%	29.41	30.00	6.19					
	Hybrid	1	150	142	6	4%	24.60	24.50	5.54	286	23.92	23.75	5.34	4.67
		2	150	144	6	4%	23.23	23.00	5.14					
	Wild	1	150	136	13	9%	15.81	15.50	5.61	275	15.61	15.25	5.51	8.33
		2	150	139	11	7%	15.40	15.00	5.41					
Carp	Farm	3	150	85	65	43%	9.50	9.30	4.58	183	10.10	10.15	4.86	39.00
		4	150	98	52	35%	10.70	11.00	5.13					
	Hybrid	3	150	111	39	26%	6.95	6.30	3.75	237	6.88	6.00	3.59	21.00
		4	150	126	24	16%	6.80	5.70	3.42					
	Wild	3	150	94	56	37%	4.82	4.20	2.36	197	4.81	4.15	2.37	34.33
		4	150	103	47	31%	4.80	4.10	2.38					
Natural	Farm	5	150	80	69	46%	5.10	5.10	2.17	191	4.71	4.70	2.15	36.33
		6	150	111	39	26%	4.42	4.40	2.09					
	Hybrid	5	150	72	78	52%	4.30	4.05	2.12	182	3.89	3.60	1.90	39.33
		6	150	110	30	20%	3.62	3.20	1.74					
	Wild	5	150	62	88	59%	2.91	2.60	1.48	145	2.93	2.60	1.40	51.67
		6	150	83	72	48%	2.94	2.60	1.37					

DNA-based parentage testing was used to identify the sampled fish back to family of origin. DNA was extracted in 96-well plates using the HotSHOT genomic DNA preparation method as recommended by manufacturers (Biotechniques, 2000). Five microsatellite markers, MHC1 [33], SSsp3016 (Genbank # AY372820), SsOsl85 [34], Ssa197 [35], and SsaF43 [36] were amplified in one PCR multiplex (PCR conditions in Additional file 1). PCR products were resolved on an ABI Applied Biosystems 3730 Genetic Analyser and sized using a 500LIZ standard (Applied Biosystems). Genemapper Version 5.0 was used to score alleles manually. Individuals were then assigned back to family using the Family Analysis Program (FAP) (v3.6) [37], an exclusion-based assignment program that has been routinely used for the purpose of parentage assignment in salmonids [8, 38, 39].

### Statistical analysis

Statistical analysis was carried out using R version 3.2.1 [40] with all critical *p*-values set to 0.05 unless otherwise stated.

#### Growth

A linear mixed model (LME) was used to investigate the effect of diet treatment, genetic background (group = farmed, hybrid, wild) and egg size on body weight at termination. The response variable was the continuous variable of log-transformed (log_10_) wet weight at termination. The full model included the fixed factor covariates of treatment and genetic group and the fixed continuous covariate of log-transformed (log_10_) and centred egg size (mean family egg diameter), plus all two-way interactions between the fixed covariates. Differences in variance patterns between the replicate treatment tanks were controlled for by including replicate nested within treatment in the model as a random intercept effect with 6 levels. Differences in variance patterns between families across the treatments were controlled for by including family nested within group as a random intercept effect (15 levels) with differing slopes for the effect of treatment.

The LME model was fitted using the *lmer* function from the *lme4* package in R [41]. Model selection of the full models was performed by the use of the *lmerTest* package, which allows for automatic model selection using the *step* function [42]. This function eliminates non-significant random effects before eliminating non-significant fixed effects using backwards selection to yield the final model. The *p*-values for the random effects are calculated using likelihood ratio tests where the significance level was set at 0.1 [42]. *P*-values for the fixed covariates, as well as the F-statistics and degrees of freedom were calculated based upon Satterthwaite’s approximations [42]. The full and final models, as given by the step function output, are presented in Table 3. Pair-wise comparisons between treatments and between groups were performed by the use of the *glht* function in the *multcomp* package [43] using the final model (Additional file 1).

**Table 3:** Model selection of the linear mixed effect model used to investigate the influence of diet treatment, genetic group and egg size upon body weight of Atlantic salmon at termination

Model	N	Response Variable	Random effects				Fixed effects					
			Variable	Chi.sq	Chi.df	*P*	Variable	Sum.sq	Num.df	Den.df	F	*P*
	1972	Log Weight	T:r	0.54	1	0.46	T x G	0.068	4	11.30	0.52	0.72
			**T/G:f**	**85.06**	**5**	**<1e-07**	G x E	0.094	2	9.22	1.46	0.28
							T x E	0.25	2	13.01	3.74	0.052
							**T**	**41.76**	**2**	**13.80**	**645.12**	**<1e07**
							**G**	**6.85**	**2**	**11.56**	**105.35**	**<1e07**
							**E**	**0.34**	**1**	**11.92**	**10.29**	**0.0076**

Significance levels of random and fixed effects included in the full LME model investigating variation in log body weight at termination. N; number of individuals. Log weight; log10 (wet weight + 1) at termination. Random effects: T:r; replicate (r) nested within treatment (T) (random intercept). T/G:f; familiy (f) nested within group (G), across treatments (T) (random intercept and slope). Chi.sq; the value of the Chi square statistics. Chi Df; the degrees of freedom for the test. P; *P*-value of the likelihood ratio test for the random effect. Fixed effects: T, diet treatment (control, carp, natural). G; genetic group (farmed, wild, hybrid). E; mean family (log10) centred egg diameter. Two-way interactions terms included in the full model: T x G, T x E and G x E. Sum.Sq; sum of squares. Num Df, numerator degrees of freedom. Den Df; denominator degrees of freedom based on Sattherwaithe’s approximations. F; F-value. The variables in bold were retained in the final model

#### Survival

A generalised linear mixed model (GLMM) was used to investigate whether diet treatment, genetic background (group = farmed, hybrid, wild) or egg size affected survival. The response variable, survival, was binary, and thus the binomial distribution was used with the default logit link function and was fitted using the Laplace approximation. The full model covariates were identical to the growth model described above. Differences in variance patterns between the replicate treatment tanks were controlled for by including replicate as a random intercept effect. Differences in variance patterns between families across the treatments were controlled for by including family as a random intercept effect with differing slopes for the effect of treatment.

The GLM model was fitted using the *glmer* function from the *lme4* package [41]. The random effect structure was investigated by fitting the full model with only one random effect at a time and plotting the 95% prediction intervals of the random effect using the *dotplot* function in the *lattice* package [44]. If all the prediction intervals of the random effect overlapped zero then this effect was removed from the final model. Backward selection using a likelihood ratio test (LRT) was performed on a full fixed effect model comparing two random effect structures (Additional file 1), i.e. a random intercept model for family versus a random intercept and slope model for family. The fixed effect structure of the final model was determined by backward selection using the *drop1* function based on AIC values [45] (Table 4). Pair-wise comparisons between diet treatments and between genetic groups were performed as for growth above [43] using the final model (Additional file 1).

**Table 4:** Model selection of the fixed effects of the generalised linear mixed model investigating mortality

		Fixed effects							
N	Response	T x G	G x E	T x E	Treatment	Group	Egg size	AIC	∆AIC
2696	Survival	x	x	x	x	x	x	2540.11	2
			x	x	x	x	x	2539.66	2.45
			x		x	x	x	2540.47	1.58
					**x**	**x**	**x**	**2542.11**	**0**
					x	x		2555.41	13.3
					x		x	2554.34	12.23
						x	x	2552.41	10.3

T x G; Treatment by group interaction. G x E; Group by egg size interaction. T x E; Treatment by egg size interaction. AIC; Akaike information criterion. ∆ AIC; difference in AIC value. Nested models which differed by less than 2 AIC were interpretted as equally good, with the simplest best fitting model chosen. The final fixed effect structure is shown in bold

## Results

### Sampling & data

The experiment was terminated after 19 weeks in week 36 of 2014 when all 1984 surviving fish were sampled. The microsatellite multiplex had an average assignment power of 99.79%, and six individuals could not be assigned unambiguously back to one family. These individuals were removed from the dataset prior to analysis. A further six individuals were removed from the dataset after being identified as outliers due to extreme condition factors, indicating recording errors during sampling. Thus, the final dataset for analysis consisted of 1972 individuals.

#### Growth

**Fig. 2:**
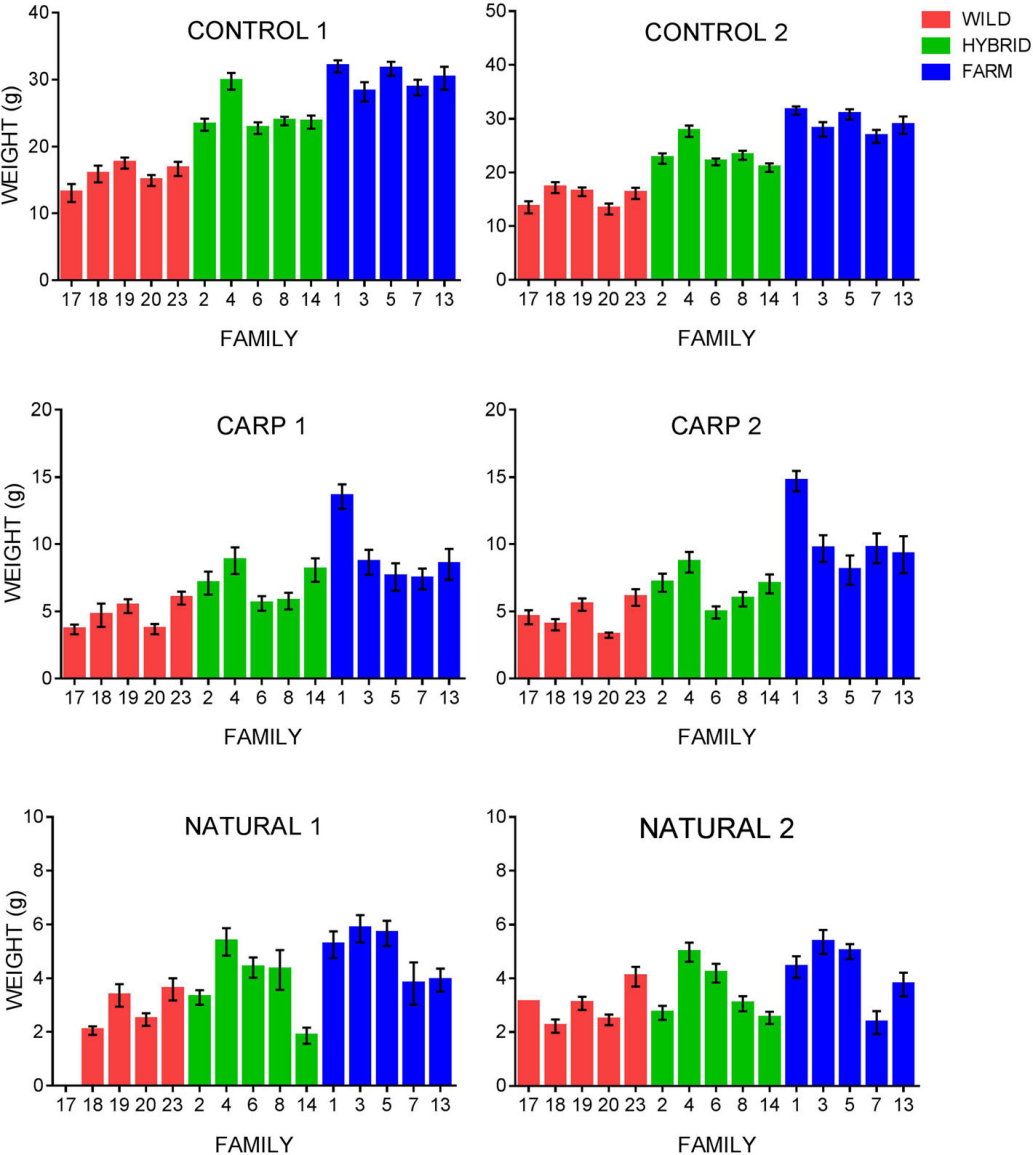
Average weight of each family within the genetic groups in the replicates of each treatment. Treatments: control, carp and natural. Weight was measured in grams and the error bars represent standard error. Farmed fish were significantly larger than hybrid and wild fish across all treatments, and family variation in growth was visible among the treatments

Overall, growth of all groups was several times higher in the control treatment in comparison with the carp and natural diet treatments: average body weight was 23.10 g in the control treatment, 7.18 g in the carp treatment and 3.92 g in the natural diet treatment. Thus, diet had a highly significant effect on growth of all groups despite the fact that the total amount of energy available to the fish in each treatment was identical (Table 3, Fig. 2).

There was a significant effect of genetic group on growth (Table 3). Across all treatments, farmed fish grew significantly larger than the hybrid fish, which were in turn larger than the wild fish (Fig. 2, Additional file 1). The relative growth difference between wild and farmed fish was highest in the carp treatment (1: 2.1), lowest in the natural diet (1:1.6) and intermediate in the control treatment (1:1.9) (Table 5). A significant interaction between treatment and group was not detected (Table 3, Fig. 4d). Thus, salmon of all genetic groups responded to the diet treatments in a similar plastic manner, resulting in similar growth reaction norms across the treatments (Fig. 4d).

**Table 5:** Relative weight differences between farmed, wild and hybrid Atlantic salmon within each diet treatment

Treatment	Group	Weight (g)	Relative difference	
			to Wild	to Hybrid
Control	Farm	29.70	1.9	1.2
	Hybrid	23.95	1.5	-
	Wild	15.55	-	-
Carp	Farm	10.14	2.1	1.5
	Hybrid	6.87	1.4	-
	Wild	4.80	-	-
Natural	Farm	4.71	1.6	1.2
	Hybrid	3.89	1.3	-
	Wild	2.93	-	-

The relative growth differences were calculated by dividing the average weight (in grams) of the farmed fish by the wild and hybrid fish respectively, and the average weight of the hybrid fish by the wild fish within each treatment

The effect of the interaction between egg size and treatment was marginally insignificant, and the effect of egg size alone was negatively correlated to weight. The latter was however due to the generally larger egg sizes of the wild families used in the present study coupled with their lower growth compared to the farmed and hybrid families. Removing the effect of egg size upon final weight in the selected LME model did not influence the results of the analysis (data not presented here). There was some visible weight variation between families within the three genetic groups, and variation between families differed furthermore between treatments (Fig. 4a-c). For instance, family 1 of farmed origin exhibited exceptional growth in the carp diet treatment in relation to the other families (Fig. 2). To control for these trends the selected LME model included family nested within group as a random intercept effect with differing slopes for the effect of treatment.

#### Survival

**Fig. 3:**
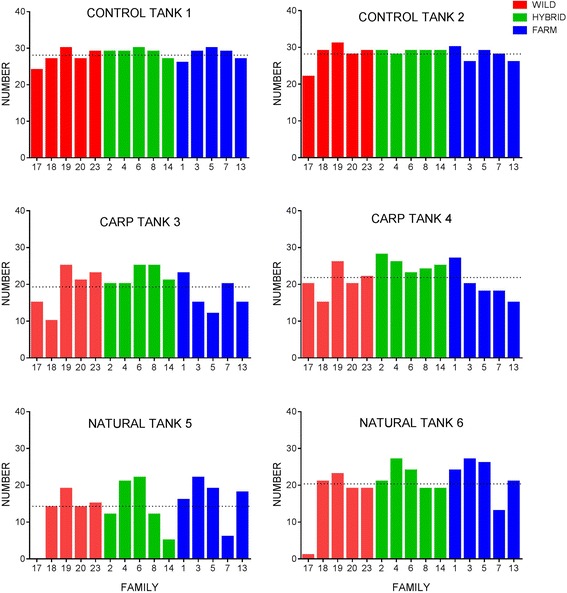
Number of fish surviving from each of the 15 families within replicates of each treatment. Treatments: control, carp and natural. Dotted horizontal lines represent the expected number of fish per family in each replicate based on average mortality

Overall survival in the control, carp and natural diet treatments was 93.78, 68.56 and 57.67%, respectively. Survival was thus highest in the control diet treatment, and was significantly different to both the carp and natural diets (Fig. 3, Additional file 1). Survival did not differ significantly between the carp and natural diet treatments, although on average survival was lower in the natural diet treatment (possibly due to the observed variation in survival between replicated tanks in this treatment) (Table 2, Additional file 1). Thus, diet had a highly significant effect on survival (Table 4). Egg size had a significant positive effect on survival (data not presented here).

**Fig. 4:**
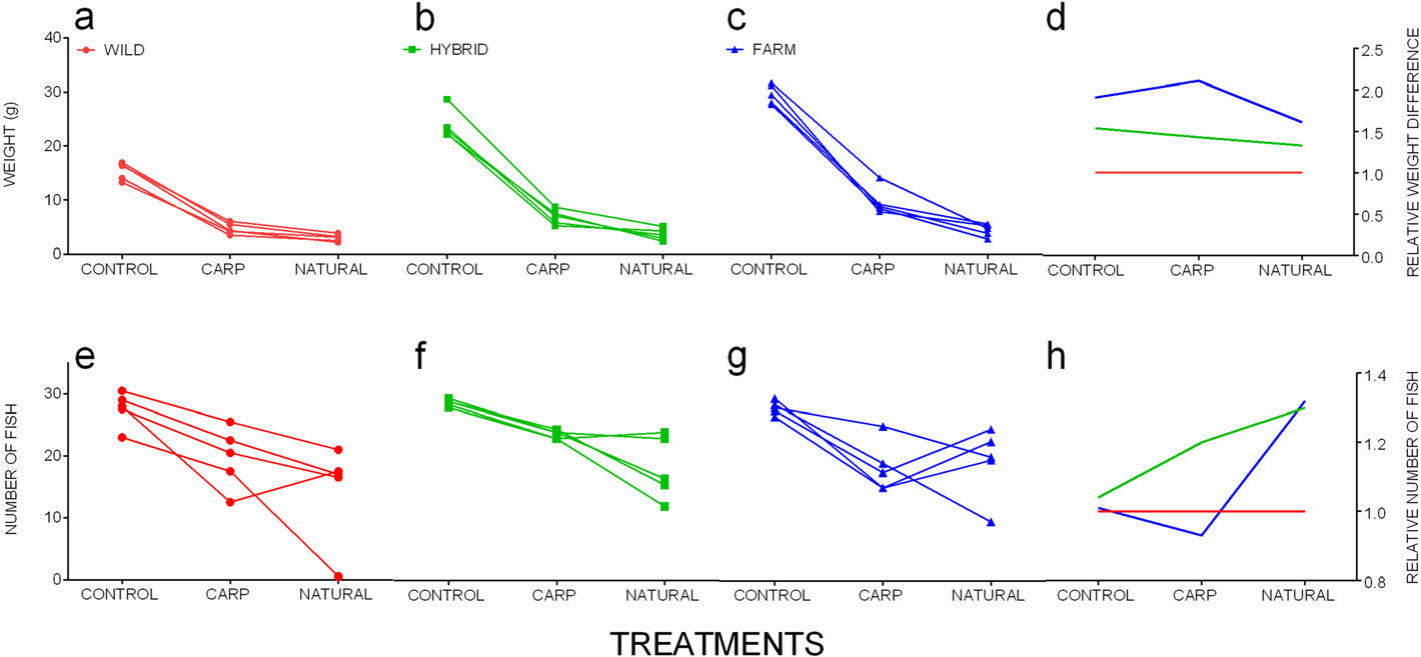
Phenotypic reaction norms for growth (**a**-**d**) and survival (**e**-**h**) across the treatments. (**a**-**c**) The phenotypic growth reaction norms for each group at the family level using untransformed weight in grams and (**d**) average weight relative to the wild group where the hybrid and farmed groups are compared to the wild group within each treatment. (**e**-**g**) The survival reaction norms for each group at the family level between the treatments and (**h**) the relative survival reaction norms for each group where farmed and hybrid fish are compared to the wild fish within each treatment. Treatments (control, carp, natural) are indicated on the x-axis

There was a significant effect of group on survival (Fig. 3, Table 4). Overall, differences in survival were not significant between hybrid and farmed fish (79 and 74% respectively) while wild fish displayed significantly lower survival to both groups (69%) (Additional file 1). Within treatments, hybrids displayed the highest average survival within the control and carp diet treatments, while the farmed fish displayed the highest average survival in the natural diet treatment (Table 2, Fig. 4h). There was no significant interaction effect between treatment and group detected (Table 4). Thus, salmon of all genetic groups responded to the diet treatments in a similar plastic manner, resulting in similar survival reaction norms across the treatments (Fig. 4e-h).

Survival differed between some of the replicated treatments tanks (Fig. 3), thus the random effect of replicate nested within treatment was retained in the final model to control for this variation. Similarly, there was an under-representation of some families, e.g., wild family 17, within the genetic groups within some of the treatments (Fig. 3) and visible variation between families within the three genetic groups, and between treatments (Fig. 4e-g). To control for this the final GLM model included family as a random intercept effect with differing slopes for the effect of treatment.

## Discussion

Farmed salmon display significantly higher growth rates than wild salmon when reared together under hatchery and commercial farming conditions, but in the wild, growth-differences between these groups are modest or marginal [7, 9, 25]. However, the mechanisms underlying this contrast between environments remain more or less completely unknown. Unravelling these mechanisms is important for our understanding of the genetic changes that have occurred in farmed salmon in response to domestication, as well as our understanding of the long term evolutionary consequences of interbreeding between farmed escapees and wild conspecifics. We hypothesised that potential adaptation of farmed salmon to a commercial diet, consisting of high-energy pellets with a specific form and nutritional profile, may contribute to the observed contrast in growth differences between the hatchery and the wild among farmed and wild salmon. To investigate this, we compared growth and survival of farmed, wild and F1 hybrid fish fed three contrasting diets under common garden hatchery conditions. Salmon of all genetic groups grew best on a commercial salmon diet, intermediate on a commercial carp diet and poorest on a natural diet. There was no interaction detected between diet and genetic group for growth, indicating that the groups all responded identically relative to each other on the different diets. Thus, similar plasticity as well as similar reaction norms towards the differing diets was detected in salmon of all origins. Similarly, all groups survived the best on the commercial salmon diet, and there was no interaction effect of diet and group for survival. Based upon these results, we find no evidence to suggest that farmed salmon have adapted to feeding on a commercial diet consisting of high-energy pellets. We therefore conclude that increased appetite represents the primary cause of farmed salmon outgrowing wild salmon when fed *ad lib* rations under hatchery conditions. In turn, it is also concluded that diet type alone, at least in the absence of other potential environmental stressors, is not the primary cause for the large contrast in growth differences between farmed and wild salmon between the hatchery and wild.

### Growth

Growth was significantly different between the treatments, being highest in the control diet, intermediate in the carp diet, and lowest in the natural diet treatment. The very large difference in overall growth between the control and carp treatment, i.e., a 68% growth decrease, occurred even though the percentage calorie density difference (MJ/kg) between the two diets was only ~15%, and that all treatments received equal total calories. The carp diet contained roughly 4.5 times as much carbohydrate, a third less protein and half as much lipid than the salmon diet. The ability of fish to utilise carbohydrates varies between species and carbohydrate complexities, and salmon are less effective at it than some other fish species [46, 47]. Commercial salmon diets typically contain low levels of carbohydrates as salmon do not require high levels of carbohydrates in their diets, unlike warm water species such as carp; although, the inclusion of low amounts of carbohydrates can facilitate the utilisation of other nutrients [47]. Farmed salmon get most of their energetic requirements from the high dietary levels of lipids and proteins [48]. Thus, it is likely that the lower growth observed in the carp treatment relative to the control diet was a result of the mismatch in the dietary levels of specific nutrients resulting in all fish not being able to fully utilise or digest the food efficiently. Previous studies have shown that a high level of dietary carbohydrate negatively affects feed utilisation and growth in several fish species, including Atlantic salmon [49], European sea bass (*Dicentrarchus labrax* L.) [50] and Wuchang bream (*Megalobrama amblycephala,* Yih 1955) [51].

Domestic selection for growth has affected various feeding related traits including appetite and FCE [10–12]. Thodesen et al. [10] found that farmed salmon consumed more food and utilised their food more efficiently than wild conspecifics under controlled conditions, and attributed this to genetic changes in domesticated fish through direct selection for growth. Similarly, Handeland et al. [11] found significantly higher growth and overall higher FCE in farmed salmon smolts compared to wild smolts under controlled conditions. In the present study, neither feed utilization nor FCE was investigated. Therefore, adaptation to nutritional content of commercial diets was indirectly tested by comparing growth of farmed and wild salmon when fed nutritionally contrasting commercial pelleted diets and a diet consisting of natural prey.

Salmon of all groups responded to the pelleted carp treatment in a similar manner, by displaying similar growth reaction norms relative to each other, between the carp and the control treatment. Thus, the effect of the pelleted carp diet, with an unfamiliar nutritional content to salmon of all origins, was similar in farmed and wild salmon, i.e., all groups displayed growth reduction of 65–71% relative to their respective growth in the control treatment. Farmed salmon utilised the commercial salmon and carp diets in the same manner relative to the wild salmon, and therefore did not utilise the familiar salmon diet better than the wild salmon. Wolters et al. [12] investigated growth of a wild and a selected strain of Canadian Atlantic salmon fed two contrasting diets consisting of either standard energy (18% fat) or high energy (32%) under controlled conditions. They detected an effect of diet on the final weight of the selected strain, where selected salmon fed the high-energy diet were 14.3% larger than selected salmon fed the standard energy diet, and no effect of diet was detected in the wild strain. The authors attribute these differences to a higher energy utilisation of the selected strain compared to the wild strain [12]. If the farmed fish in the present study were adapted to the nutritional content of commercial salmon pellets and therefore utilised it better than the wild fish, then we would expect the relative growth divergence between farmed and wild fish in the carp diet to be lower than in the control treatment, as this diet contained a nutritional content to which none of the strains could possibly have been adapted to. The present study therefore found no evidence that farmed fish have become adapted to the nutritional content of the commercial salmon pellets per se, as they were not able to utilise it better than the pelleted carp diet.

Growth of salmon is generally found to be less under natural than domestic conditions (although see [52]). Growth is strongly associated with water temperature [53], and growth is also linked to the metabolic costs associated with actively seeking prey, defending territories, predator avoidance, and the abundance of food and energy in river systems. As the present study took place within a hatchery with no predation, food was not limiting nor did fish have to actively seek prey, it is unlikely that the lower overall growth in the natural diet treatment, i.e., an 83% growth decrease, is attributable to any of the above. While efforts were made to ensure that the natural diet contained a similar calorie content to the other diets, it is possible that fish were unable to obtain and utilise the correct balance of nutrients to maximise growth. Or put simply, it is possible that fish were unable to consume enough of this moisture rich food to match the calorie content of the two formulated diets and this restricted their growth. As above, the farmed, hybrid and wild salmon displayed similar reaction norms for growth between the control treatment and the natural diet treatment i.e., all groups displayed growth reduction of 83–84% relative to their respective growth in the control treatment. If farmed salmon are adapted to the form of commercial diets, or if wild salmon simply just won’t eat pellets in the same manner as farmed salmon, one would expect the relative growth differences between farmed and wild salmon to be significantly lower when fed a natural diet as compared to a pelleted diet. The present study therefore found no evidence that farmed fish are unable to maintain their relative growth advantage with a natural diet. Whether the growth differences observed between farmed and wild salmon, on all diets tested here, were due to farmed salmon displaying an increased appetite or due to an overall increased utilisation of feed regardless of form and content, cannot be disentangled however. While several fold differences in growth between farmed and wild salmon under hatchery conditions have been thoroughly documented in the literature [9], only modest changes in feed utilization have been suggested thus far [10]. It is suggested that appetite could be the major driving force towards the observed growth differences between farmed and wild salmon when feed at *ad lib* rations.

Farmed salmon escaping into the wild may not initially be accustomed to actively seeking and selecting prey due to differences in environmental experiences relative to wild salmon. Release experiments have demonstrated that farmed salmon previously reared on pellets were less likely to actively feed than their wild conspecifics in a natural environment, and were more likely to ingest prey of lower nutritional value [54]. In general, after a period of acclimation farmed fish display similar feeding behaviour as their wild conspecifics, although this often depends on the life stage [55]. However, experiments conducted in the wild from the egg stage reveal that the diets of the offspring of farmed and wild salmon overlap [24, 25]; and so farmed fish are able to feed in the wild. In the present study, the natural diet was composed of dead organisms; therefore, it is possible that the natural diet was too accessible to the fish, and using a live diet where the fish had to chase the prey itself, may have elicited a different response between the salmon groups. Live prey was not used as we would not be able to disentangle if a possible reduction in growth difference between farmed and wild salmon would be due to farmed salmon being adapted to the commercial diet, or due to farmed salmon not being able to catch live prey. This however, could form the basis of a future study.

Although the absolute growth differences observed between the farmed, hybrid and wild salmon experimental groups in the present study are lower than previously observed under hatchery conditions [8, 9], it is clear that multiple generations of selection have resulted in farmed salmon which outgrow their wild conspecifics, although this effect is not as pronounced in the wild. In the present study, the hybrids originated from maternal farmed and paternal wild crosses and therefore, hybrid growth may be influenced by maternal effects [56]. However, hybrids in the present study displayed somewhat intermediate growth, similar to findings of other comparative studies [8, 57, 58], illustrating that additive inheritance is responsible for the majority of the variation of this trait.

### Survival

Studies show that fish which have been reared in captivity and fed only commercial diets display a low survival in the wild once they are released or escape as they are not initially able to efficiently switch from pelleted feed to natural feed [55, 59, 60]. Comparative survival studies in the wild found that the freshwater survival of farmed fish was low compared to wild conspecifics, and that hybrids generally displayed intermediate survival [61, 62]. Skaala et al [25] observed that offspring of farmed fish planted out as eggs in a natural river system had a significantly reduced survival relative to their hybrid and wild conspecifics. Similarly, Fleming et al. [24] found that offspring of farmed fish had lower early stage survival than wild conspecifics in the wild, although at a later stage (parr to smolt) there was no difference in survival. Among other things, lower survival in farmed salmon may be the result of inefficient feeding behaviour [54, 55] and behavioural differences, such as increased aggression or decreased predator awareness [57, 63], which may also expose fish of farmed backgrounds to more predation than their wild conspecifics. Farmed fish may also have become adapted to the form and nutritional content of commercial salmon diets and consequently lost their ability to feed in the wild, contributing to their low survival in nature. If farmed salmon had lost some ability to digest natural feed, it would be expected that they would display the lowest survival in the natural treatment. However farmed salmon displayed the highest average survival in the natural treatment. Therefore, there was no evidence to suggest that farmed fish have become adapted to the form and nutritional content of commercial salmon diets to the extent it influences the survival of their offspring when fed exclusively on a natural diet mimicking that available in the wild. Indeed, as above, studies have demonstrated that the diet composition of farmed salmon in the wild tends to overlap with those of wild salmon [24, 25].

It is possible that the lower survival within the natural and carp diet treatments relative to the control treatment is due to all fish being unable to efficiently utilise the diets or consume enough calories as discussed above. Within the natural diet treatment wild fish had the lowest average survival. Sundt-Hansen et al. [64] found that offspring of farmed salmon displaced and out-competed offspring of wild salmon in a short-term experiment conducted in a simulated stream environment, resulting in a lower survival of wild conspecifics. In the present study, food was presented in excess in each treatment to reduce or eliminate resource competition. It is still theoretically possible that farmed and hybrid fish in the natural treatment may have gained a competitive size advantage over the wild salmon. However, a study looking into growth of farmed, hybrid and wild salmon when reared communally (as in the present study), or in single strain tanks, found no evidence for a competitive interaction between the strains (i.e., the relative difference between the groups was identical despite being communally and singly-reared) [9]. Potentially the acceptability of the non-live prey may have influenced the palatability of the natural diet for the wild fish. Hybrid fish exhibited particularly high survival in the carp treatment relative to their farmed and wild conspecifics. It is unknown why there was such a large difference in survival relative to their parental groups in the carp treatment.

Egg size was significant and positively correlated with survival, suggesting that a larger egg size was beneficial for survival under these conditions. Studies indicate that egg size has a positive effect on survival in salmonids [65, 66], which may explain why wild fish in the present study had larger eggs on average than their conspecifics. In two of the treatments in the present study wild fish survived the worst on average, despite having larger egg sizes than the farmed salmon, which indicates that the wild exhibited an even lower than expected survival.

## Conclusion

The present study provides insights regarding the potential genetic changes that have occurred in salmon in response to domestication, and the potential mechanisms underpinning genetic and ecological interactions between farmed escapees and wild salmonids following interbreeding in the wild [27–29, 67]. Understanding the impacts of growth differences between farmed, hybrid and wild fish is important for conservation and management of wild fish, in addition to the sustainable development of the aquaculture industry. The present study was unable to find evidence that the elevated growth differences observed between farmed and wild salmon in the hatchery is a result of farmed fish being adapted to commercial salmon diets, i.e., either nutritional content or form. Similarly, we were unable to find evidence that farmed salmon perform less well on an *ad lib* diet containing organisms which are typically present in the wild, relative to wild salmon. Overall these results indicate that increased appetite is the primary reason why farmed salmon display increased growth rates, as compared to wild salmon, under *ad lib* feeding conditions. Our study took place in a hatchery environment, did not include live prey, nor took predation or other environmental parameters which may influence growth and survival into account. Therefore, we encourage further studies under wild or semi-natural conditions to elucidate why farmed salmon do not outgrow wild salmon extensively in the natural environment.

## Additional file

Additional file 1: Table S1. Experimental crosses. **Table S2.** Approximate nutritional content of each diet. **Table S3.** PCR conditions for the microsatellite multiplex used to assign individuals back to family. **Table S4.** Multiple comparisons for overall growth for both groups and treatments using a Tukey adjustment for multiple comparisons. SE; standard error. **Table S5.** Model selection of the random effect of the generalized linear mixed effect model used to investigate survival. **Table S6.** Multiple comparisons for overall mortality for both groups and treatments using a Tukey adjustment for multiple comparisons. SE; standard error. (DOCX 25 kb)
