# Supplementary material for: Plasticity in growth of farmed and wild Atlantic salmon: is the increased growth rate of farmed salmon caused by evolutionary adaptations to the commercial diet?
Source: BMC Evol Biol. 2016 Dec 1;16:264. doi: 10.1186/s12862-016-0841-7 (PMC5134087; doi:10.1186/s12862-016-0841-7)
Supplement: Additional file 1: Table S1. — Experimental crosses. Table S2. Approximate nutritional content of each diet. Table S3. PCR conditions for the microsatellite multiplex used to assign individuals back to family. Table S4. Multiple comparisons for overall growth for both groups and treatments using a Tukey adjustment for multiple comparisons. SE; standard error. Table S5. Model selection of the random effect of the generalized linear mixed effect model used to investigate survival. Table S6. Multiple comparisons for overall mortality for both groups and treatments using a Tukey adjustment for multiple comparisons. SE; standard error. (DOCX 25 kb) [file 12862_2016_841_MOESM1_ESM.docx]

**Additional tables**

Table S1: Experimental crosses.

| Family | Female | Group | Male | Group | Family type |
| --- | --- | --- | --- | --- | --- |
| 1 | M1 | Mowi farm | M9 | Mowi farm | Farm |
| 2 | M1 | Mowi farm | E11 | Etne wild | Hybrid |
| 3 | M2 | Mowi farm | M10 | Mowi farm | Farm |
| 4 | M2 | Mowi farm | E12 | Etne wild | Hybrid |
| 5 | M3 | Mowi farm | M11 | Mowi farm | Farm |
| 6 | M3 | Mowi farm | E13 | Etne wild | Hybrid |
| 7 | M4 | Mowi farm | M12 | Mowi farm | Farm |
| 8 | M4 | Mowi farm | E14 | Etne wild | Hybrid |
| 13 | M7 | Mowi farm | M15 | Mowi farm | Farm |
| 14 | M7 | Mowi farm | E17 | Etne wild | Hybrid |
| 17 | E1 | Etne wild | E11 | Etne wild | Wild |
| 18 | E2 | Etne wild | E12 | Etne wild | Wild |
| 19 | E3 | Etne wild | E13 | Etne wild | Wild |
| 20 | E4 | Etne wild | E14 | Etne wild | Wild |
| 23 | E7 | Etne wild | E17 | Etne wild | Wild |

Three different populations were used to make three experimental groups: 5 farmed families of the Mowi commercial strain; 5 F1 hybrid families; and 5 wild families of the Etne strain. F1 hybrid families were created by crossing a farmed female with a wild male and each hybrid family is thus a maternal and paternal half-sib to a respective farmed and wild family.

Table S2: Approximate nutritional content of each diet.

|  | Salmon  pellets | Carp  pellets | Natural ingredients | | | |
| --- | --- | --- | --- | --- | --- | --- |
|  |  |  | Cyclops | Daphnia | Black  mosquito  larvae | Glassworm  (white mosquito  larvae) |
| Protein | 55 | 29 | 3.5 | 2.4 | 5 | 5 |
| Fat | 18 | 10 | 0.4 | 0.7 | 1 | 1 |
| NFE (Carbs) | 8.7 | 46.5 | 0.4 | 0.3 | 0.9 | 0.9 |
| Ash | 10.5 | 5 | 0.1 | 0.7 | 0.8 | 0.8 |
| Other (Moisture) | 0.5 | 7.5 | 95.3 | 96.3 | 92 | 92 |
| Energy content | 21.6 MJ/kg | 18.6 MJ/kg | 0.78 MJ/kg | 0.70 MJ/kg | 1.31 MJ/kg | 1.31 MJ/kg |

The nutritional and energy content of the commercial pelleted diets were obtained from the manufacturer (Skretting), and the nutritional content of the invertebrate ingredients of the natural diet was obtained from the manufacturer (Ruto Frozen Fishfood) and energy content was calculated manually.

Table S3: PCR conditions for the microsatellite multiplex used to assign individuals back to family

|  | Temperature (°C) | Time |  |
| --- | --- | --- | --- |
| Denaturation | 94 | 4 min |  |
| Denaturation | 94 | 50 s | Repeat x 26 |
| Annealing | 55 | 50 s |  |
| Extension | 72 | 80 s |  |
| Final extension | 72 | 10 min |  |
| Storage | 4 | unlimited |  |

Table S4: Multiple comparisons for overall growth for both groups and treatments using a Tukey adjustment for multiple comparisons. SE; standard error.

| Comparisons | Estimate | SE | Z value | P value |
| --- | --- | --- | --- | --- |
| Control - Carp | 0.55 | 0.021 | 26.41 | <1e-10 |
| Natural - Carp | -0.28 | 0.035 | -7.89 | <1e-10 |
| Natural - Control | -0.83 | 0.030 | -27.18 | <1e-10 |
| Hybrid - Farm | -0.079 | 0.021 | -3.83 | 0.00036 |
| Wild - Farm | -0.33 | 0.023 | -14.32 | <1e-04 |
| Wild - Hybrid | -0.25 | 0.023 | -10.56 | <1e-04 |

Table S5: Model selection of the random effect of the generalized linear mixed effect model used to investigate survival.

|  |  | Random effects | | |  |  |  |  |  |  |  |  |
| --- | --- | --- | --- | --- | --- | --- | --- | --- | --- | --- | --- | --- |
| N | Response | T:t | F | T:F | Df | AIC | BIC | logLik | Deviance | Chisq | Chi Df | P |
| 2696 | Survival | x | x |  | 16 | 2558.6 | 2653 | -1263.3 | 2526.6 |  |  |  |
|  |  | x | x | x | 21 | 2540.1 | 2664 | -1249 | 2498.1 | 28.53 | 5 | 29e-05 |

T:t; replicate nested within treatments (random intercept). F; family (random intercept). T:F; family across treatments (random intercept and slope). Df; Degrees of freedom. AIC; Akaike information criterion. BIC; Bayesian information criterion; logLik; loglikelihood value. Chisq; Chi square value. Chi Df; Chi square degrees of freedom. P; p-value.

Table S6: Multiple comparisons for overall mortality for both groups and treatments using a Tukey adjustment for multiple comparisons. SE; standard error.

| Comparisons | Estimate | SE | Z value | P value |
| --- | --- | --- | --- | --- |
| Control - Carp | 2.35 | 0.50 | 4.66 | <1e-04 |
| Natural - Carp | 0.40 | 0.46 | 0.86 | 0.45 |
| Natural - Control | -1.95 | 0.51 | -3.84 | <1e-04 |
| Hybrid - Farm | 0.89 | 0.33 | 2.69 | 0.11 |
| Wild - Farm | 0.074 | 0.34 | 0.22 | 0.01 |
| Wild - Hybrid | -0.81 | 0.36 | -2.29 | <0.001 |
